# Oral Squamous Cell Carcinoma Mimicking Lichenoid Reaction After Implant Placement: A Case Report

**DOI:** 10.7759/cureus.50804

**Published:** 2023-12-19

**Authors:** Hamad Albagieh, Shaima E Alabdulkareem, Wajd Alharbi, Shahd M Alqahtani, Ghayda Algoblan

**Affiliations:** 1 Dentistry, College of Dentistry, King Saud University, Riyadh, SAU

**Keywords:** erythroleukoplakia, gingival overgrowth, lichenoid contact reaction, dental implant, oral squamous cell carcinoma

## Abstract

The early detection of oral squamous cell carcinoma (OSCC) poses significant challenges, especially if it mimics a benign condition. This report presents a case of a 79-year-old nonsmoker Saudi male patient with an alveolar lesion that initially resembled an implant-induced reaction but upon biopsy revealed dysplastic squamous epithelium indicative of squamous cell carcinoma (SCC). This case highlights that lesion mimicry, the absence of pain, and low cancer awareness can cause diagnostic delays. Treatment options for OSCC include surgery, chemotherapy, and radiotherapy, with surgery being the primary treatment modality. This case emphasizes the need for heightened vigilance among healthcare providers, regular follow-ups, and enhanced cancer awareness to promote early detection and intervention. Recognizing the diverse clinical presentations of OSCC remains essential for effective management and improved patient outcomes, despite the complexities of its etiology and diagnostic challenges.

## Introduction

Over 90% of oral malignancies are caused by squamous cell carcinoma (SCC) [1]. Oral cancer is the third most common malignancy in Saudi Arabia, behind lymphoma and leukemia. Up to 26% of all head and neck cancers found in Saudi Arabia each year are oral cancers [2]. Patients with oral squamous cell carcinoma (OSCC) present as older males with a delay in seeking professional medical attention typically. The delay tends to be more common among individuals from low socioeconomic backgrounds [1]. Oral squamous cell carcinoma (OSCC) is responsible for at least two deaths out of every 100,000 cases in the Middle East, according to a report released by the World Health Organization (WHO) [3,4].

The delay in seeking professional care may be due to the minimal pain experienced in the early growth phase. A biopsy may not be performed until weeks or months have passed due to the absence of a high index of suspicion for oral squamous cell carcinoma (OSCC) among healthcare providers [1].

Oral squamous cell carcinoma is a complex malignancy with multifactorial etiology, which suggests that a combination of factors rather than a single causative agent is likely responsible for its development. This involves the interplay of extrinsic and intrinsic factors. Extrinsic factors, such as tobacco use and alcohol consumption, have been well established as significant contributors to OSCC. Malnutrition and iron-deficiency anemia are examples of OSCC intrinsic factors. However, heritable factors do not seem to play a significant role in the etiology of squamous cell carcinoma [1].

In Saudi Arabia, the tongue is the most common site for oral cancer [2]. Interestingly, gingival and alveolar carcinomas, although less associated with tobacco smoking, display a distinctive propensity to mimic benign lesions, leading to challenges in diagnosis. This mimicry often results in delayed recognition, with the lesions being misinterpreted as inflammatory or reactive conditions, such as pyogenic granuloma or periodontal disease [1].

Furthermore, metastasis is not an early occurrence in oral cavity carcinomas, yet due to delayed diagnoses, a significant proportion of patients present with cervical metastases at the time of diagnosis, further highlighting the criticality of early detection [1].

It is crucial to note that many oral squamous cell carcinomas have been documented to be associated with or preceded by a precancerous lesion, especially leukoplakia, underscoring the significance of vigilance and the thorough evaluation of suspicious oral lesions even when initially presenting as seemingly benign conditions [1].

This paper presents a non-tobacco-related case of OSCC of the lower alveolus mimicking an implant-induced lichenoid reaction, emphasizing the importance of recognizing and distinguishing between potentially malignant lesions and benign conditions for prompt diagnosis and appropriate management.

## Case presentation

**Figure 1:**
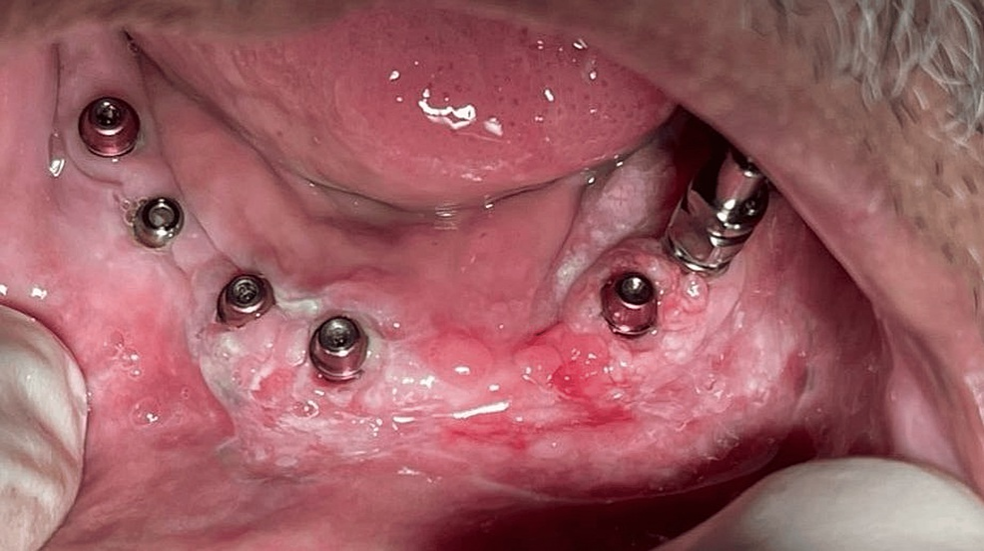
Intraoral photo showing the first lesion that appeared in October 2022 and was diagnosed with implant-induced mucositis.

A 79-year-old Saudi male patient presented to the Department of Oral Medicine and Radiology at King Saud University Hospital, Riyadh, Saudi Arabia, with the chief complaint of pain in his mouth that has been present for the past four months. One year prior, he had undergone full-arch implant placement for the construction of an overdenture. Following the implant placement, he developed a lesion around the implants and was diagnosed with implant-induced mucositis (Figure 1). He received corticosteroid treatment, which resulted in healing. Subsequently, his prosthesis was placed on the implants. After one month, a new lesion developed in the same region, and the patient reported self-administering corticosteroid mouthwash believing it might elevate the symptoms. However, he reported no improvement.

The patient is a nonsmoker and does not drink alcohol. He is hypertensive and takes aspirin and Concor 5 mg once daily. He takes omeprazole for heartburn. He has a history of spinal surgery without complications, abdominal hernia repair without complications, and prostate cancer surgery without chemotherapy or radiation therapy. He is currently not taking any medication for his prostate cancer. He has no relevant family history and is not known to be allergic to any specific drugs, foods, or chemicals.

**Figure 2:**
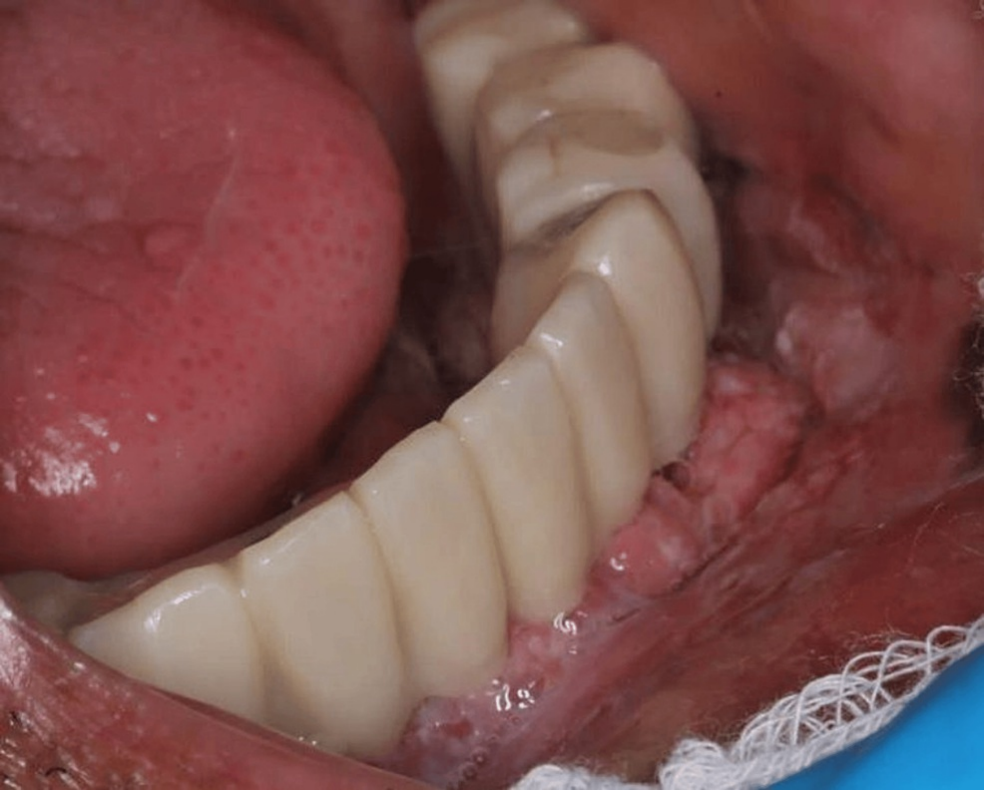
After one year, exophytic verrucous papillary red and white intraoral lesion has appeared on the lower alveolar ridge extending from the region of #43 to #35.

**Figure 3:**
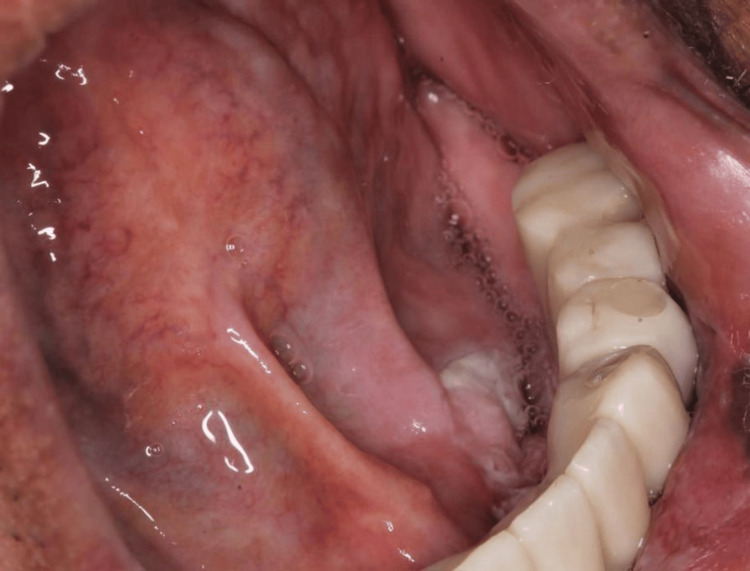
Lingual extension of the lesion toward the floor of the mouth.

The intraoral examination revealed an exophytic verrucous papillary lesion on the lower alveolar ridge from the region of #43 to the region of #35, extending to the lingual side (Figures 2, 3). This lesion can resemble verrucous erythroleukoplakia (red and white lesion). Eventually, it developed into a necrotic ulcer with irregular, elevated, and indurated borders.

**Figure 4:**
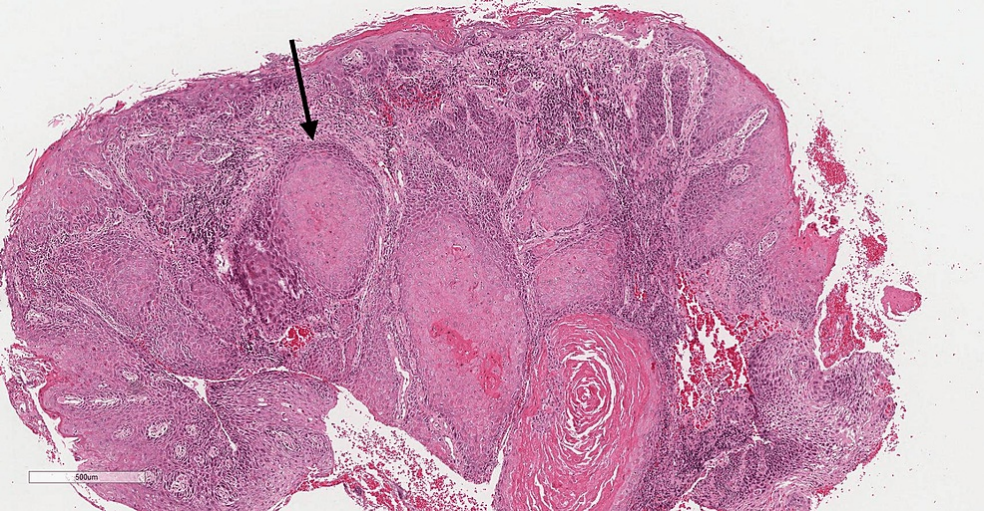
Hematoxylin and eosin (H&E) low-power magnification demonstrating dysplastic surface epithelium and islands of malignant epithelial cells (black arrow). Power of magnification: 10×

**Figure 5:**
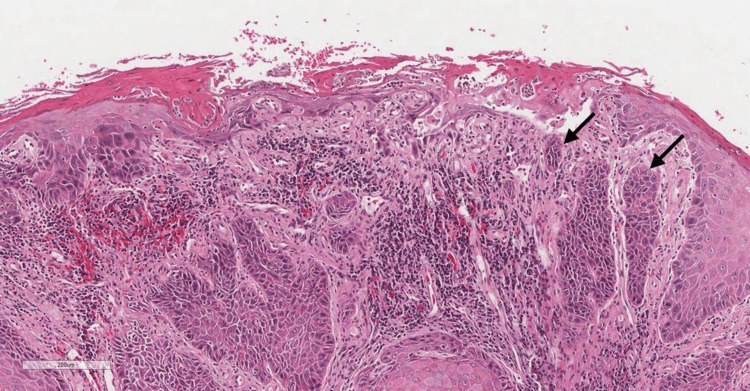
Hematoxylin and eosin (H&E) high-power magnification demonstrating dysplastic features of the epithelium such as nuclear and cellular pleomorphism, dyskeratosis, increased N-C ratio, and hyperchromatic nuclei (black arrows). Power of magnification: 25× N-C: nuclear-cytoplasmic

After two weeks of follow-up, an incisional biopsy was performed. Under magnifications of 10× and 25×, the microscopic features revealed dysplastic squamous epithelium covering a severely inflamed lamina propria. The epithelial rete ridges demonstrate prominent endophytic and convoluted growth. Moreover, malignant epithelial cells invaded the lamina propria. The epithelium also shows dysplastic features such as nuclear and cellular pleomorphism, dyskeratosis, increased nuclear-cytoplasmic (N-C) ratio, hyperchromatic nuclei, and keratin pearl formation. Areas show the loss of basement membrane integrity and single-cell infiltration (Figures 4, 5).

**Figure 6:**
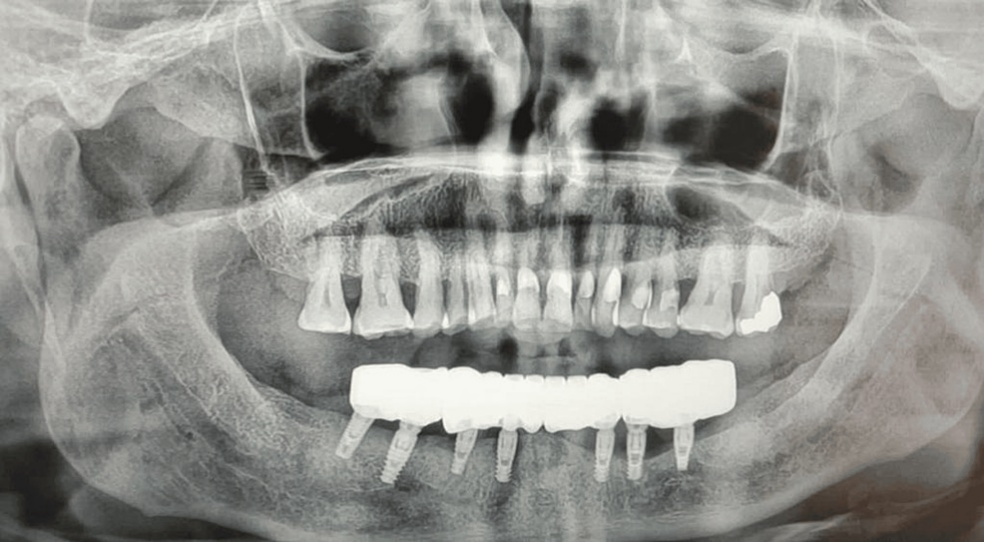
Panoramic radiograph showing advanced horizontal bone loss around mandibular left implants.

A panoramic radiograph was taken as a part of routine prosthodontics treatment showing advanced horizontal bone loss around the mandibular left implants after the prosthesis placement (Figure 6).

Following histopathological testing, the patient was diagnosed with oral squamous cell carcinoma. It should be mentioned that this is a primary neoplasm, and it is not related to the previous prostate cancer.

The nature of oral squamous cell carcinoma and the patient's overall health influence the choice of a particular treatment approach. The specific affected area, the size and extent of the lesion, and distance metastasis are all important factors when it comes to carcinoma. The factors related to the patient are the age, overall medical conditions, and history of treated OSCC [5]. Treatment options for OSCC are numerous and consist of surgical removal whether it is excision or resection, chemotherapy, radiotherapy, photodynamic therapy, epidermal growth factor receptor (EGFR) inhibitors, and a combination of these, either simultaneously or in a specific order [6,7]. Surgery is still the most initial and definitive treatment option for most of the patients with OSCC [5]. In this case, the treatment plan is surgical removal and then follow-up visits to check the recovery of the patient.

In Saudi Arabia, 56% of SCC patients experienced recurrence within five years following their initial treatment. This high recurrence rate might be linked to late detection and intervention [3].

## Discussion

Cancer poses an increasing global health challenge, the dominant cause of death in developed nations and the second leading cause in developing nations [8]. Although there is significant progress in various therapeutic interventions, oral cancer remains a life-threatening condition with minimal improvement in prognosis and survival. The primary reason could be attributed to delayed detection or misdiagnosis.

The primary etiologic factors associated with oral squamous cell carcinoma are smoking and smokeless tobacco. "Shammah," also known as a form of smokeless tobacco, was identified in a study located in Jazan, Saudi Arabia, as the most important predictable variable for OSCC. It was also seen that the combined use of risk factors such as shammah, shisha, and cigarettes has serious implications for the onset of OSCC [4]. Other risk factors in Saudi Arabia include obesity, genetics, sedentary lifestyle, viral infection, and iodine and vitamin D deficiency [9]. The patient, in this case, did not have any apparent potential risk factors for oral squamous cell carcinoma.

An estimated 50% of all OSCC cases are located in the posterior lateral border of the tongue, and this has the highest incidence of OSCC [10], in contrast to our case, which was in the alveolar ridge around the dental implants.

Oral lichenoid lesions can be provoked by a hypersensitivity response to different dental materials such as metals, acrylates, flavorings, and many other types of allergens. Allergy reactions in peri-implant tissue can be induced by titanium. A similar case was reported by du Preez et al.; in the postoperative period following implant installation, a foreign body reaction was associated with signs and symptoms of severe local irritation and patient discomfort. After the implant was removed, complete healing was achieved [11]. Oral lichenoid lesions were considered to have more treatment resistance and a high malignant transformation rate [12]. Therefore, it is essential to conduct careful monitoring and close follow-up of potentially malignant disorders to detect early malignant transformation.

The use of dental implants is increasing; hence, there must be a comprehensive understanding of their impact on the host immune system, their biological contribution to the formation of chronic inflammation, and their diverse clinical presentations [13]. Previous literature has shown that the number of reported cases of OSCC in association with dental implants has increased over time [14]. Most malignant lesions that were reported were misdiagnosed as peri-implantitis [15,16]. A study by Agha-Hosseini and Rohani suggests that the chronic inflammation caused by dental implants might be the reason for OSCC in some cases [13]. The review of the literature has been inconclusive if dental implants themselves can develop cancer; however, it can be stated that implants may act as an irritant and/or inflammatory trigger [17].

This is a case of OSCC development in a patient at low risk for oral cancer. The carcinoma presented in the lower alveolar ridge around the area of the implants that had been placed one year ago. Comparing to the six cases reported in the systematic review of implant-related OSCC cases conducted by Brabyn et al. between 2008 and 2017, of these, only one case resembles our study in terms of risk factors [14].

This is a rare case of oral squamous cell carcinoma that is mimicking lichenoid reaction in association with dental implants. Further research must be conducted about the relation of oral squamous cell carcinoma to peri-implant tissue as this contributes to early diagnosis and effective intervention that significantly enhance patient outcomes.

## Conclusions

This case highlights the challenge of identifying oral squamous cell carcinoma and distinguishing it from benign conditions. It emphasizes the need for vigilance among healthcare providers, especially when lesions mimic reactive conditions. Despite not involving traditional risk factors such as smoking or alcohol consumption, this case shows the complex nature of OSCC's etiology. It underscores the importance of regular follow-ups and heightened awareness to facilitate early detection. Ultimately, it highlights the critical need for a comprehensive understanding of OSCC's clinical presentations to ensure timely interventions and better patient outcomes.
